# Combination of Manuka Honey and Chitosan-Based Biomaterial for the Treatment of Dehisced Wound: Case Report

**DOI:** 10.3390/life16081279

**Published:** 2026-08-01

**Authors:** Zuzana Šufliarska, Katarína Vdoviaková, Lenka Krešáková, Ján Danko, Pavol Rusnák, Ľubomír Medvecký, Mária Giretová, Jozef Bíreš, Filip Humeník, Jana Pobehová

**Affiliations:** 1 Department of Morphological Sciences, University of Veterinary Medicine and Pharmacy in Košice, 041 81 Košice, Slovakia; sufliarska.zuzicka@gmail.com (Z.Š.); katarina.vdoviakova@uvlf.sk (K.V.); lenka.kresakova@uvlf.sk (L.K.); jan.danko@uvlf.sk (J.D.); pavol.rusnak@nke.agel.sk (P.R.); lmedvecky@saske.sk (Ľ.M.); filip.humenik@gmail.com (F.H.); 2 PET Point Veterinary Clinic, 984 01 Lučenec, Slovakia; 3 Division of Functional and Hybrid Systems, Institute of Materials Research of SAS, 040 01 Košice, Slovakia; mgiretova@saske.sk; 4 Clinic of Ruminants, University of Veterinary Medicine and Pharmacy in Košice, 041 81 Košice, Slovakia; jozef.bires@uvlf.sk; 5 Clinic of Burns and Reconstructive Medicine, AGEL Teaching Hospital, 040 15 Košice-Šaca, Slovakia; 6 Faculty of Medicine, Pavol Jozef Šafárik Univeristy in Košice, SNP 1, 040 11 Košice, Slovakia; 7 East Slovak Institute of Cardiovascular Diseases, Ondavská 8, 040 01 Košice, Slovakia

**Keywords:** wound healing, dehiscence, manuka honey, chitosan, biomaterial

## Abstract

**Background**: Surgical wound dehiscence (SWD) is defined as a complete or partial separation of the surgical incision site after surgery and represents a serious complication in soft tissue surgery. **Case presentation**: We present a clinical case of wound dehiscence in a 7-month-old dachshund. The primary cause was an attack by another dog, leading to the development of a deep bite wound in the back area perforating the anus and numerous minor injuries in the same area. After initial treatment and apparently good general health, numerous necrotic foci were observed during wound cleaning on the 3rd day after treatment. The wound was subsequently revised and an experimentally developed biomaterial based on chitosan in combination with manuka honey was applied. The use of this combination resulted in an accelerated healing process and almost complete closure of the wound already on the 12th day after application. **Conclusions**: This case demonstrates and simultaneously points to the promising possibilities of using chitosan-based biomaterials and honey as natural products for the successful treatment of wounds and management of postoperative complications.

## 1. Introduction

The present day is associated with numerous incidents that carry a high risk of injury, resulting in various types of wounds or burns [1]. Despite the daily progress made in medicine, we increasingly observe situations where even simple wounds develop complications. These can lead to impaired healing, patient hospitalization, and, in severe cases, the amputation of the affected limb or even a fatal outcome [2]. For this reason, it is necessary to develop solutions that are cost-effective, rapid, and efficient. A combination of new biomaterials, natural products and cell therapy are among those options [3]. This combination may exhibit excellent physicochemical, structural and mechanical properties, would be well accepted by the body, and would accelerate and improve wound healing [4,5]. All mentioned attributes should be met in the final stage by a chitosan-based biomaterial enriched with honey, which we have developed and tested. Chitosan, as a natural compound, exhibits antimicrobial activity, improves hemostatic effect, promotes cell proliferation and mediates complete wound regeneration and epithelial reconstruction [6]. In the phase of hemostasis, it promotes platelet aggregation and, conversely, prevents fibrinolysis and accumulation of erythrocytes due to its positively charged -NH_2_ group [7,8]. In addition to the properties already mentioned, chitosan has anti-inflammatory and analgesic effects. The anti-inflammatory activity of chitosan can be associated with its binding to CD14, TLR4 and CR3 receptors on macrophages together with its slight upregulation of the expression of IL-10 and TGF-β1 [9,10]. The analgesic effect of chitosan is achieved by reducing the levels of inflammatory mediators at the site of injury and by absorbing ions released from the site of inflammation [11]. In the phase of proliferation, the ability to stimulate the release of platelet-derived growth factor (PDGF) and transforming growth factor (TGF) is utilized. Chitosan precipitation through the dermis may aid in the healing of injured cells by creating a system that connects cells and stimulates collagen production while maintaining adequate oxygen penetration, which aids the remodeling phase of wound healing [12].

Natural products that show a positive effect in wound healing is becoming increasingly popular. One such natural substance is honey. People have been using honey for thousands of years to heal wounds [13]. The role of honey in the wound healing process lies mainly in its antimicrobial effects, unique pH, but also in its ability to influence the osmotic pressure in the wound tissue. Natural honey, as a product of bees, is a viscous liquid with a very low water activity (a_w_ < 0.91). Chemically, honey is largely composed of simple sugars such as glucose (22–41%) and fructose (27–44%) with small amounts of other sugars such as galactose or maltose. Honey also contains other components such as proteins or enzymes [14]. However, it is important to emphasize that the detailed composition of honey varies depending on the type of honey and the conditions in which the specific bee colony is located [15].

The therapeutic potential of honey lies precisely in its composition, since the low water activity and high sugar content create an environment that has a strong antimicrobial effect. After application to the surface of the wound, an environment unsuitable for bacterial growth is created [16]. However, the specific antibacterial activity of non-peroxide honey is related to the presence of glyoxal, 3-deoxyglucosulose, and methylglyoxal (MGO), compounds that are present in the highest amounts in manuka honey [17]. MGO even shows a high antimicrobial effect against pathogens such as methicillin-resistant Staphylococcus aureus strains, vancomycin-resistant enterococci, and Pseudomonas aeruginosa [18].

Honey for healing also acts as an immunomodulator and chemoattractant in wound healing. Immunomodulatory properties of honey were linked to suppressing the TLR4/NF-κB signaling pathway, leading to lower secretion levels of key pro-inflammatory cytokines, including TNF-α, IL-1β, IL-6, CXCL2 and CCL2. Additionally, honey inhibits the activity of cyclooxygenases 1 and 2 (COX1 and COX2), which are involved in prostaglandin synthesis. During the healing process, honey also influences cell migration, especially keratinocytes, through the Def-1 molecule [19,20].

Due to the influence of osmotic pressure, the natural removal of metabolic waste products from the wound to the surface occurs. Furthermore, it helps transport nutrients and oxygen from the deep layers to the wound itself. In addition, the low pH of honey increases tissue oxygenation, while free radicals, which lead to tissue damage, are removed by flavonoids and aromatic acids [21,22]. By covering the wound, a layer is created that prevents drying out and further deepening of the wound. Honey exhibits a preventive effect against eschar formation by blocking the conversion of plasminogen to plasmin, an enzyme that breaks down fibrin within the wound without affecting the collagen matrix [23]. The present study describes the positive impact of chitosan-based biomaterial in combination with Manuka honey in the therapy of a complicated dehisced wound in a dog patient.

## 2. Materials and Methods

### 2.1. Material Preparation

PHB (Goodfellow, Cambridge, UK)/chitosan scaffolds (Sigma-Aldrich, St. Louis, MO, USA) were prepared by mixing PHB (2% (*w*/*v*) solution in propylene carbonate) and chitosan solutions (1% (*w*/*v*) acetic acid solution) using a magnetic stirrer at 400 rpm. The mass ratio of PHB:chitosan was 1:1. The PHB/chitosan suspension was obtained by precipitation of polymers after adding acetone (Sigma-Aldrich, for analysis) and NH_3_ (aq, 25%, Fluka, Seelze, Germany) and 3D-printed forms (width × length × inner height = 5 × 10 × 0.4 cm plates) were filled with suspension after washing with distilled water and filtering. The samples were frozen at −20 °C and lyophilized (lyophilizer, ilShin Biobase Europe, Ede, The Netherlands) for 8 h, with the open porosity of the PHB/chitosan scaffolds being 90 ± 3%. The surface of lyophilized samples was coated with a hydrogel layer composed of agarose and gelatin in mass ratio equal to 2:1. The final biopolymer composite scaffolds (GEL/PHBCHIT) were sterilized in an autoclave at 121 °C.

### 2.2. Microstructure Characterization

The microstructure of prepared wet GEL/PHBCHIT samples and dry lyophilized scaffolds was observed with an inverted optical fluorescence microscope (Leica DM IL LED, Heerbrugg, Switzerland) equipped with a CCD camera in the VIS mode at ×50 magnification and by scanning electron microscope (JEOL FM SEM JSM-7000F, JEOL Ltd., Akishima, Japan) after coating with gold, respectively.

The changes in hydrophilicity of the lyophilized samples were analyzed by measuring the contact angle between surfaces of water drop and dry samples, which were prepared by pressing PHB/chitosan scaffolds, powder PHB and chitosan at 90 MPa in a steel mold to achieve the same surface textures almost free of pores. Static contact angle measurements were performed using the sessile drop method. A total of 10 µL of distilled water was gently positioned on the surface of samples using a microsyringe and the contact angles at the liquid–solid interface were determined by image analysis of digital photographs using Image J8 software. The mean value was calculated from three measurements.

### 2.3. Manuka Honey Characterization

Manuka honey used for the treatment of a specific case was analyzed for the content of proteins, flavonoids, polyphenols, and sugars. For the analysis of proteins, we used the Coomassie blue method together with SDS PAGE gel electrophoresis on polyacrylamide gels. The total amount of polyphenols in honey was determined by using Folin–Ciocalteu reagent (FC). The flavonoid content in the honey was analyzed using a method based on the formation of complexes with AlCl_3_. HPLC chromatography was used for deciphering phenolic compound profiles, sugar content as well as the representation of individual saccharides in honey. The analysis procedure is described in detail in a previously published study [23].

### 2.4. Application of Manuka Honey and GEL/PHBCHIT Biomaterial for Wound Healing

As the honey component for wound therapy management, we used commercially available sterile 100% manuka honey (*Leptospermum scoparium*) from New Zealand (Kruuse Manuka G, Langeskov, Denmark) in the concentration 0.15 mL/cm^2^.

### 2.5. Case Description

**Figure 1:**
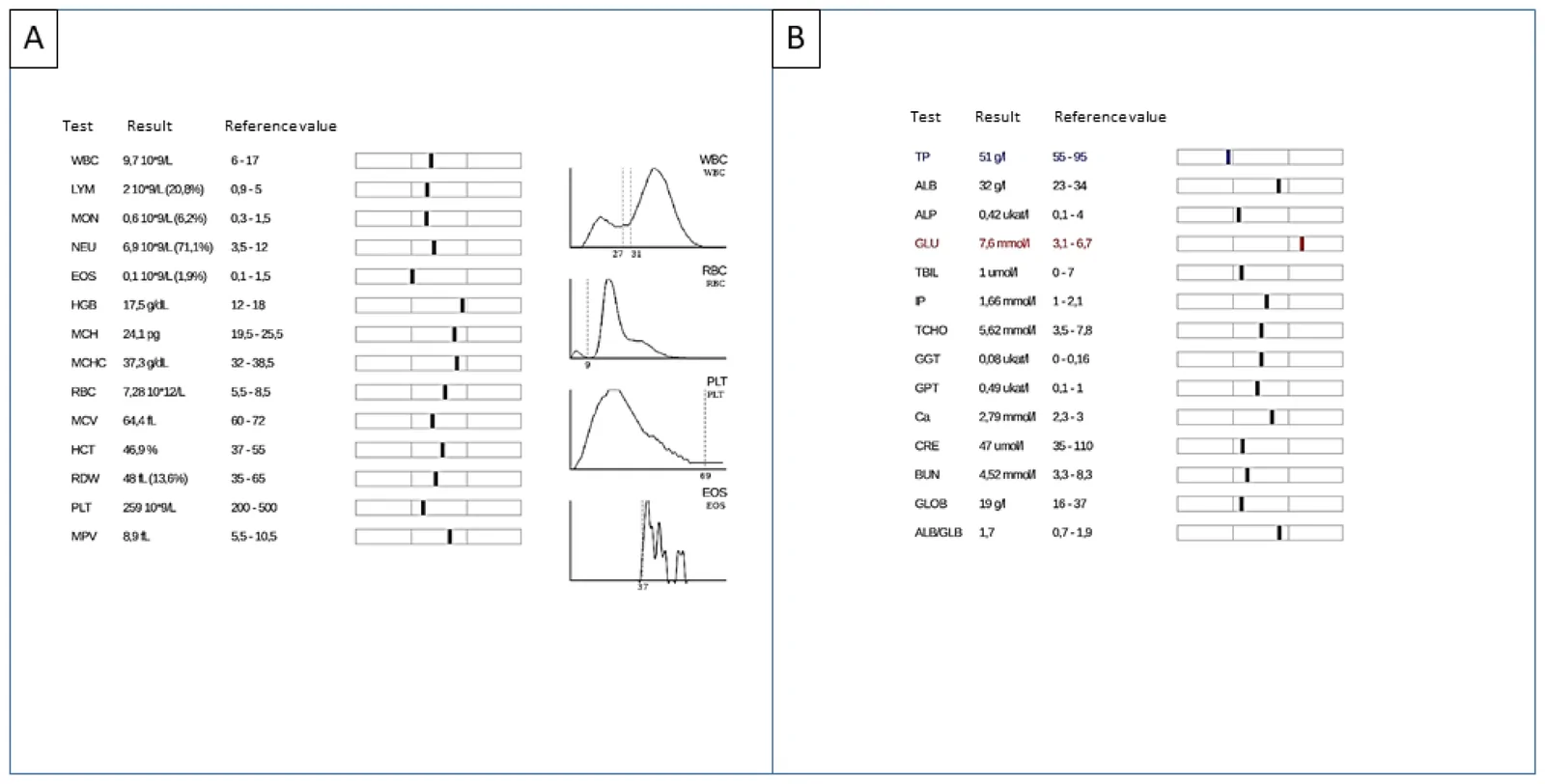
Complete results of laboratory assessment of hematological (**A**) and biochemical (**B**) parameters on the clinic admission day. All examined parameters were in physiological range. Slightly increased glycemia values were observed—7.6 mmol/L; reference value 3.1–6.7 mmol/L.

The patient (female dog, dachshund, 7 months old, 5 kg) was admitted to the veterinary clinic after being attacked by another dog. As a result of the attack and subsequent fight, 1 large wound (12 × 6 cm) and many smaller bite wounds were present in the gluteal region and at the base of the tail on the right side of the body. Clinical examination revealed the following triage values: body temperature 38.5 °C, respiratory rate—33 per minute, heart rate 140 beats per minute (BPM), CRT 1–2 s. The lung field examined by auscultation showed no pathological findings. Hematological and biochemical parameters were normal (Figure 1). X-ray examination also concluded with no pathological findings. Wound inspection and subsequent digital rectal examination revealed a perforation of the rectal wall (approximately 2 cm) and the presence of feces within the wound.

Due to the severity of the condition, surgery was indicated to repair the intestinal wall as well as to repair the soft tissues in the injured area. Before the initiation of sedation, the patient was preventively administered the antiemetic Maropitant at a dose of 1 mg/kg (Cerenia, Zoetis, Parsippany, NJ, USA). Butorphanol (Butomidor, VetViva Richter GmbH, Welse, Austria) dosage 0.2 mg/kg and diazepam (Alzane, Laboratorios SYVA S.A.U., Avda, León, Spain) dosage 0.2 mg/kg were administered intravenously to sedate the patient prior to the operation. For the induction of anesthesia, propofol (Propofol MCT/LCT 1% Fresenius inj. inf., Fresenius Kabi Austria GmbH, Graz, Austria) in a dosage of 4 mg/kg was used. Further prolongation of anesthesia was carried out by isoflurane (Isoflurin 1000 mg/g liq. inh., VETPHARMA ANIMAL HEALTH, S.L., Barcelona, Spain), dosage MAC 1.5–2.0%. The surgical site was prepared according to the principles of sterility and asepsis.

During the surgical procedure, wound revision, removal of necrotic parts and extension of the wound edges were performed. Polydioxanone absorbable monofilament of 3/0 thickness (Kruuse PD-X Suture, USP 3-0/EP 2, Langeskov, Denmark) was used for rectal suture. Subsequently, a drain was inserted into the wound and removed in the tail area. Polydioxanone absorbable monofilament of 2/0 thickness (Kruuse PD-X Suture, USP 2-0/EP 3, Langeskov, Denmark) was used for subcutaneous suture and non-absorbable monofilament of the same thickness (Daclon, SMI A.G., Vith, Belgium) was used for skin suture. At the lowest point, the wound was left open for free drainage of secretions. As further drug therapy, antibiotics (metronidazole (Efloran, KRKA, Novo Mesto, Slovenia)) at a dose of 10 mg/kg every twelve hours and NSAIDs (metamizole (40 mg/kg, Novalgin, Sanofi Aventis, Paris, France)) were administered.

**Figure 2:**
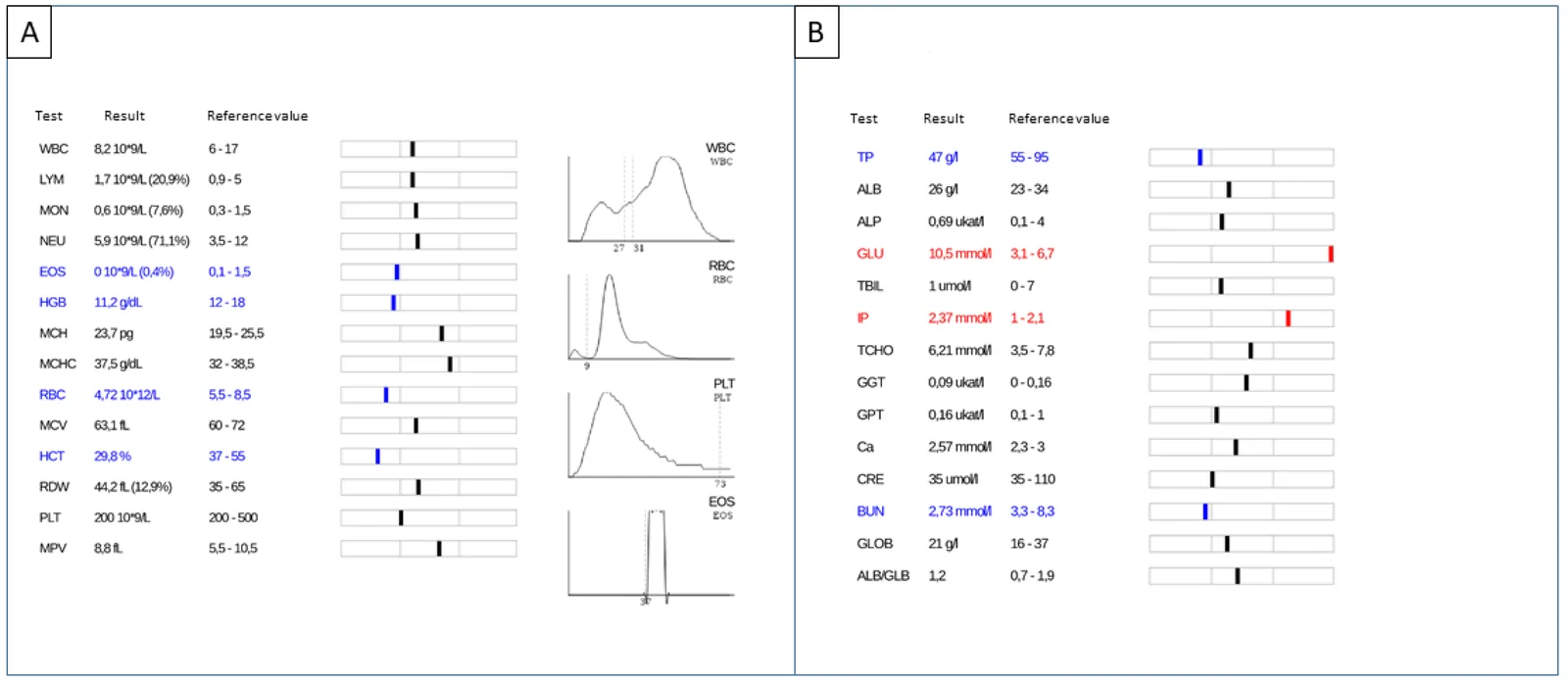
Complete results of laboratory assessment of hematological (**A**) and biochemical (**B**) parameters on the 3rd day after surgery. A complete blood count revealed mild anemia (HGB: 11.2 g/dL, physiological range: 12—18 g/dL; erythrocytes: 4.72 × 10^12^/L, physiological range: 5.5–8.5 × 10^12^/L; hematocrit: 29.8%, physiological range: 37–55%). Biochemical parameters showed hyperglycemia (10.5 mmol/L, physiological range: 3.1—6.7 mmol/L) and elevated inorganic phosphorus levels (2.37 mmol/L, physiological range: 1.0—2.1 mmol/L).

Continuous health check-up was performed every 12 h during hospitalization. The wound was checked on the 3rd day after surgery, when increased pain in the patient was observed. At the same time, changes in the blood count and biochemical indicators were detected (Figure 2). Blood count showed mild anemia (HGB—11.2 g/dL (physiological range 12–18 g/dL); erythrocytes—4.72 × 10^12^/L (physiological range 5.5–8.5 × 10^12^/L); hematocrit 29.8% (physiological range 37–55%)). Biochemical parameters showed high glycemia (10.5 mmol/L, physiological range 3.1–6.7 mmol/L) and elevated inorganic phosphorus levels (2.37 mmol/L, physiological range 1.0–2.1 mmol/L).

**Figure 3:**
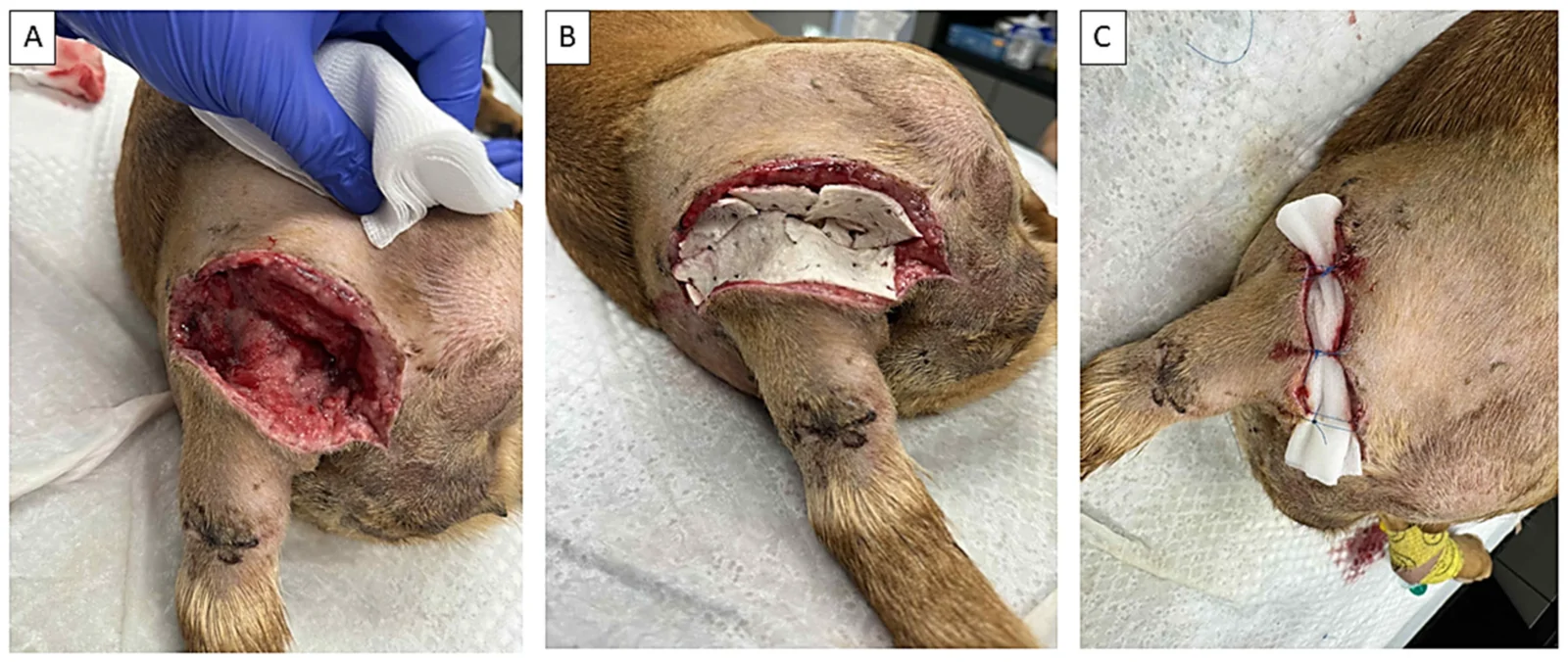
Surgical intervention after detection of wound dehiscence. Wound after complete revision and necrotization (**A**), application of GEL/PHBCHIT biomaterial in combination with manuka honey (**B**), and securing the biomaterial (**C**).

Subsequently, necrotic foci were detected during wound inspection. Due to the overall pain, the patient was sedated and a wound revision was performed under full anesthesia, during which the drain was removed and a necrectomy was performed. Because of the failure of the conventional approach and the large extent of wound damage, a chitosan-based biomaterial was applied together with manuka honey and further treatment management was guided by approximation of the wound edges (Figure 3). Wound care and inspection were performed every 72 h and at each examination, 1–1.5 mL of manuka honey was applied on the wound surface.

## 3. Results

### 3.1. Preparation and Characterization of Chitosan-Based Biomaterial

**Figure 4:**
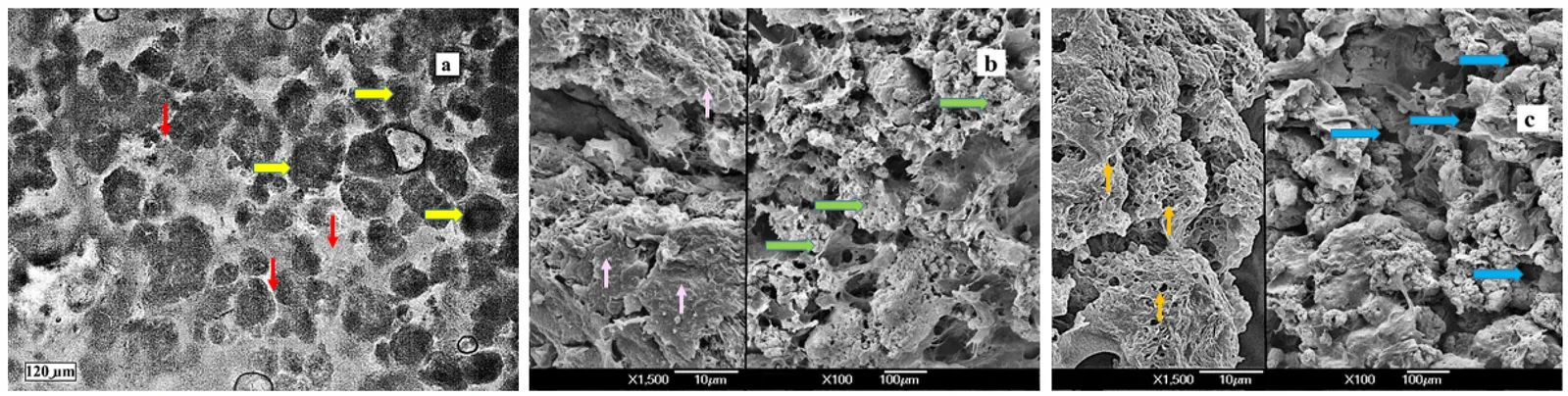
Microstructure of GEL/PHBCHIT biomaterial. Globular agglomerates of PHB nanoparticles (yellow arrows) were detected in hydrogel matrix, and chitosan microfibers (red arrows) almost completely disappeared from the microstructure of GEL/PHBCHIT scaffolds (**a**). Separated chitosan microfibers connected to the plate-like clusters (pink arrows) and agarose fiber clusters that entrapped globular agglomerates of PHB particles (green arrows) (**b**). Changes in the porosity (micropores are represented by an orange arrow and macropores are represented by a blue arrow) of the scaffolds after lyophilization of the hydrogel-containing samples (**c**).

A more detailed analysis of the physicochemical properties of GEL/PHBCHIT reported in our previously published article [24] showed a decrease in the average molecular weight of polyhydroxybutyrate and chitosan biopolymers after sterilization, as well as a decrease in the number of larger macropores (>400 μm) with a simultaneous increase in the number of pores with a size of <100 μm due to filling with a relatively viscous agarose/gelatin hydrogel (Figure 1) and swelling of chitosan. Globular agglomerates of PHB nanoparticles were detected in the hydrogel matrix, and chitosan microfibers almost completely disappeared from the microstructure of the GEL/PHBCHIT scaffolds (Figure 4a). Furthermore, in the microstructures of lyophilized scaffolds, separated chitosan microfibers, mostly connected to the plate-like objects in the PHB/chitosan samples, were almost completely integrated and mixed with the agarose fibers into plate-like clusters that entrapped globular agglomerates of PHB particles, as seen in Figure 4b,c, while changes in the macroporosity of the scaffolds were found after lyophilization of the hydrogel-containing samples (Figure 4b,c).

### 3.2. Manuka Honey Characterization

A detailed chemical profile of manuka honey is provided in a previously published study [23]. Briefly, the total protein content in the manuka honey utilized in the study was 0.13 ± 0.02 wt%. The identified proteins are represented by the major royal jelly proteins with Mw~48–70 kDa and proteins originating from nectars or pollen between Mw 20 and 30 kDa. The total polyphenol and flavonoid contents of the manuka honey were 1.45 mg/kg and 0.87 mg/kg. Detected polyphenols isolated from sample of manuka honey were luteolin (8.2 ± 1 µg/g), chrysin (55.6 ± 6 µg/g), gallic acid (44.8 ± 7 µg/g), kaempferol (18.1 ± 2 µg/g), 3,4,5-trimethoxybenzoic acid (26.3 ± 3 µg/g), chlorogenic acid (162.1 ± 11 µg/g), rutin (27.5 ± 5 µg/g), quercetin (7.5 ± 1 µg/g), ferulic acid (26.8 ± 3 5 µg/g), coumaric acid (76.0 ± 6 µg/g), syringic acid (37.9 ± 5 µg/g), methylsyringate (37.0 ± 4 µg/g), caffeic acid (68.2 ± 8 µg/g), protocatechuic acid (36.7 ± 3 µg/g). Regarding saccharides, the presence of 4 saccharides was detected—fructose (50.1 ± 2 wt%), glucose (27.5 ± 1 wt%), saccharose (3.8 ± 0.5 wt%) and maltose (5.7 ± 0.8 wt%).

### 3.3. Application of GEL/PHBCHIT Material in the Treatment of Dehiscing Wounds

**Figure 5:**
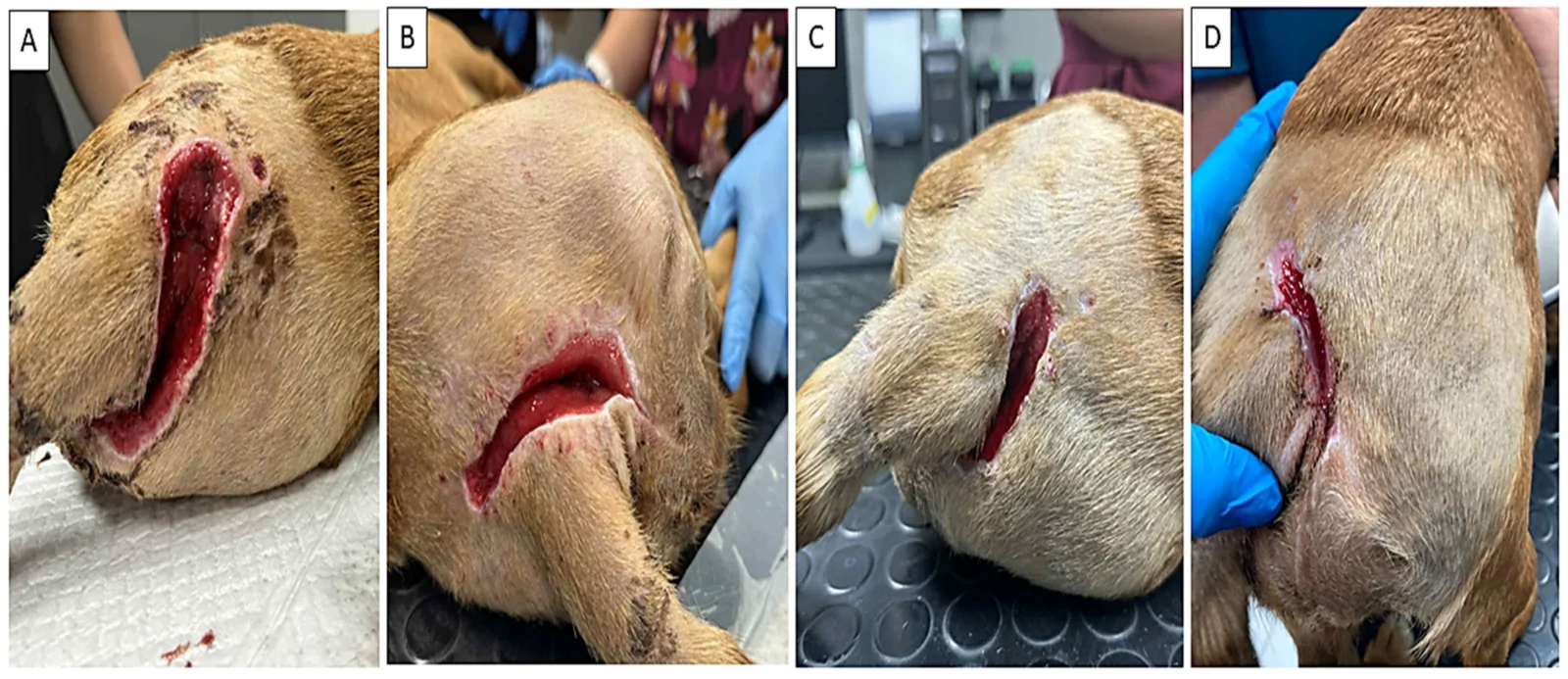
Wound healing process after application of GEL/PHBCHIT biomaterial combined with manuka honey. Representative photo documentation of healing process after biomaterial enriched with manuka honey at 3rd (**A**), 6th (**B**), 9th (**C**), and 12th (**D**) days of treatment.

After necrectomy and surgical revision of the dehisced wound, we applied GEL/PHBCHIT biomaterial to the wound in combination with sterile 100% manuka honey (*Leptospermum scoparium*) from New Zealand (Kruuse Manuka G, Langeskov, Denmark) in an amount of 0.15 mL per cm^2^ of biomaterial. Already after 72 h of biomaterial application, the chitosan-based biomaterial was almost completely resorbed, swelling decreased, and no significant wound secretion was observed. Almost complete wound closure occurred on the 12th day after application (Figure 5D).

## 4. Discussion

The main goal of the presented case study was to point out the positive effect of an innovative chitosan-based biomaterial with the addition of gel and manuka honey on the healing of dehisced wounds. Dehiscence can be characterized as a partial or complete separation of previously approximate wound edges due to failure of proper wound healing. This scenario typically occurs 5 to 8 days after the surgery, when healing is still in its early stages. Main causes of dehiscence include ischemia, infection, increased abdominal pressure, diabetes, or malnutrition [25]. A non-negligible factor in a dehiscent wound is a biofilm. A biofilm can be characterized as a complex, organized colony of bacteria that adheres to the wound bed and forms a protective sheath [26]. In the case of dehiscence (breakdown of the wound edges), it creates an impermeable barrier. This structure protects bacteria from antibiotics and the immune system, thereby preventing healing and maintaining the wound in chronic inflammation [27]. The mechanism of this pathology is based on the production of bacterial toxins and enzymes (e.g., proteases), which break down newly formed tissue and collagen, thus preventing wound re-growth. Bacteria protected by a biofilm are up to 1000 times more resistant to antibiotics and conventional disinfection than bacteria in the normal external environment. An open dehiscent wound is thus constantly exposed to external contamination, which accelerates the re-formation of the biofilm [28]. Honey, with its strong antimicrobial properties, finds application in this case as well. Honey is a highly effective, natural agent against wound biofilms. When applied to a wound dehiscence, medical-grade honey penetrates the protective biofilm matrix, disrupts bacterial communication, and kills embedded pathogens—ultimately accelerating healing and reducing the need for antibiotics or re-suturing [29,30].

For the treatment of our specific case, a dehisced bite wound in a young dog, a chitosan-based biomaterial was used, as previously characterized in detail in a published article, in combination with medicinal manuka honey [24].

The innovation of the biomaterial consisted in the addition of a gelling component consisting of a mixture of agarose + gelatin in a ratio of 2:1. The impetus for the addition of the gel itself resulted from clinical requirements based on the improvement of the material properties regarding easier manipulation. The gelling component adds elasticity and strength to the original material. The improvement of biological properties, which play a significant role in wound healing, is also significant and not negligible [31]. It should be noted that all scaffolds support cell proliferation on the surfaces. For good cell adhesion, proliferation, optimal adsorption of proteins or other substances on the surface of the scaffolds, and suitable flux of nutrients distributed with body fluids as aqueous solutions through the microporous structure of the scaffolds (in addition to the pore size distribution), wettability is a very important parameter that can be characterized by the contact angle [32]. The contact angle of PHB and the original chitosan after compression was 83 ± 2° and 73 ± 1.5°, which are higher values than 62 ± 2° found for lyophilized PHB/chitosan and GEL/PHBCHIT scaffolds. It was shown that the optimal contact angle for promoting fibroblast proliferation on the surface of the substrates should be around 60° [33]. Such a significant decrease in the contact angle of the composite scaffolds was due to the presence of an enhanced amount of water adsorbed on the large specific surface of the microfibers or nanoparticles in the precipitated biopolymers after lyophilization, as well as a higher proportion of the amorphous phase, which is able to bind water in micro- and nanopores. It should be noted that the contact angles between a water droplet and the surface of chitosan or composite samples decrease rapidly with time due to swelling, and wet composites containing hydrogels are inherently fully wettable by water or aqueous media, including body fluids [34]. The question is the possible thermal instability of natural polymers containing chitosan and gelatin and recommend the use of non-thermal sterilization methods. Thermosterilization (autoclaving) of chitosan biomaterials is scientifically accepted if the correct form of the material is chosen. Scientific studies confirm that while autoclaving chitosan in solution leads to its strong degradation, sterilization of dry chitosan powder (flakes) or finished physical hydrogel does not damage the properties of the biomaterial and preserves its biocompatibility [35,36].

Chitosan participates in all 4 phases of the healing process—hemostasis, inflammation, proliferation and remodeling [37]. In the hemostatic phase, it promotes hemagglutination and platelet adhesion through the interaction between the positively charged -NH_2_ groups and the membranes of erythrocytes and platelets, which have negative polarity [38,39]. In the inflammatory phase of wound healing, chitosan acts as an immunomodulator, by inducing the release of various pro- and anti-inflammatory cytokines, chemokines, growth factors and bioactive lipids from innate immune cells [40]. In the proliferation phase, chitosan promotes neovascularization and angiogenesis by promoting the production of VEGF and IL-8 as proangiogenic factors [7,41]. Chitosan precipitation through the dermis can aid in the healing of injured cells by creating a system that connects cells and stimulates support production while maintaining adequate oxygen penetration. In addition, it is biodegradable, biocompatible, hemostatic, and has anti-inflammatory and antibacterial properties. Chitosan is beneficial for exudate absorption and promotes tissue rejuvenation as well as the formation of essential skin fibers, thus directly helping to manage the remodeling phase [42].

Honey has been used as a therapeutic agent for wound healing for thousands of years. Sources refer to its use in wound healing as far back as ancient Egypt, Rome, and Greece [43,44,45]. Honey promotes wound healing through its antibacterial, anti-inflammatory, and antioxidant properties, while also helping to keep the wound moist and clean [46]. Currently, medical-grade honey is mainly used in medicine, due to its precisely defined composition and ability to ensure sterility, which is essential for wound healing [47].

However, there are currently arguments for the simultaneous use of chitosan-based biomaterials and medicinal honey in wound healing. The main role of chitosan is to form a strong but flexible skeleton and network for cell growth and the formation of new tissue, while simultaneously maintaining the applied honey at the site of application. On the other hand, honey is used in this combination as a bioactive substance thanks to all the above-mentioned properties [48,49]. Thanks to the above-mentioned facts of both natural products, chitosan and honey, their use in wound healing is justified, because the combination results in a product where the hemostatic, regenerative and antibacterial properties of chitosan are applied together with the immunomodulatory and antibacterial properties of honey. Our results are in line with this. The primary cause of failure of conventional therapy was apparently bacterial infection, resulting in wound dehiscence and the formation of numerous necrotic foci. The results of the hematological examination, in which we observed eosinopenia, also point to bacterial infection in this case. The occurrence of eosinophil values at the lower limit with the possibility of further decrease and development of eosinopenia in bacterial wound infections has also been described by other authors [50,51]. In our case, we observed relatively high blood glucose levels during the initial examination and during the follow-up examination. Elevated blood glucose levels are very common following severe injuries, burns, and wounds. Hyperglycemia in wounds is caused by the body’s stress response, which is triggered by injury and infection, leading to a surge in hormones that increase blood sugar [52]. However, what was striking in our case is the fact that already on the 3rd day after the application of chitosan-based biomaterial in combination with manuka honey, there was a decrease in pain, swelling and pathological secretion at the wound site. Macroscopically, the wound showed physiological progress, including high blood supply to the wound base, wound contraction and approximation of the wound edges, which resulted in almost complete wound closure on the 12th day after the start of therapy without any need for further antibiotic treatment or repeated surgical intervention. Similar effects of chitosan, honey or hydrogels have been described by other authors, but with significantly smaller wounds in in vivo conditions on animal models of mice or rats [53,54,55].

Based on the clinical view, the question arises whether the given positive effect was not caused by the use of antibiotic therapy or NSAIDs. Let us therefore try to explain the above conjectures. Nonsteroidal anti-inflammatory drugs (NSAIDs) can delay wound healing. By inhibiting the COX pathways, they reduce the production of key prostaglandins (like PGE_2_) needed for tissue repair [56]. This suppression can lead to decreased keratinocyte proliferation, reduced vascular endothelial growth factor (VEGF) expression, and impaired angiogenesis [57]. The primary use of antibiotic therapy (metronidazole-based antibiotics) is standard in cases of anaerobic infections, which are also associated with disruption of the gastrointestinal tract [58]. Metronidazole is primarily an antibiotic for anaerobic bacteria and protozoa infections, but it also exerts distinct immunomodulatory and anti-inflammatory effects. It reduces inflammation by inhibiting neutrophil activity, decreasing the production of pro-inflammatory cytokines (such as IL-1β and TNF-α), and acting as an antioxidant. On the other hand, its epithelization and tissue-rebuilding potential is also applied in the wound healing process [59,60]. Considering the wound healing process and the development of the pathological process in the described case, we believe that the use of the above-mentioned conventional procedures did not bring the expected positive effect on wound healing.

Considering all the above facts, the combination of chitosan and honey shows promise in the relatively cost-effective and simple treatment of wound complications, especially when multidrug-resistant strains of bacteria are increasingly common. However, due to limitations such as only one described case, the absence of a control group, the young age of the individual, good condition and health status, as well as care, it is necessary to handle the obtained results with caution, and further, more extensive studies are needed to definitively confirm the effectiveness of the given procedure.

## 5. Conclusions

The presented results of the clinical case show a positive effect of the chitosan-based biomaterial and honey combination, both natural products in the therapy of wound complications and complicated wounds. These findings represent a promising future for the use of these management practices in wound treatment, and after positive results from large-scale studies, the treatment procedures could be applied in wider veterinary and human surgical practice.

## Data Availability

The data presented in this study are available upon request from the corresponding author.
